# Prevalence of Parent-Reported Food Allergy in a Mexican Pre-School Population

**DOI:** 10.3390/jcm12155095

**Published:** 2023-08-03

**Authors:** Jesús Gilberto Arámburo-Gálvez, Oscar Gerardo Figueroa-Salcido, Giovanni Isaí Ramírez-Torres, Elí Terán-Cabanillas, Martina Hilda Gracia-Valenzuela, Aldo Alejandro Arvizu-Flores, Cesar Antonio Sánchez-Cárdenas, José Antonio Mora-Melgem, Luisamaria Valdez-Zavala, Feliznando Isidro Cárdenas-Torres, Noé Ontiveros

**Affiliations:** 1 Nutrition Sciences Postgraduate Program, Faculty of Nutrition Sciences, Autonomous University of Sinaloa, Culiacan 80010, Mexico; gilberto.aramburo@uas.edu.mx (J.G.A.-G.); oscar.figueroa@uas.edu.mx (O.G.F.-S.); eteranc@uas.edu.mx (E.T.-C.); cesar.sanchez@uas.edu.mx (C.A.S.-C.); josemora.uacng@uas.edu.mx (J.A.M.-M.); luisamariavaldez13@gmail.com (L.V.-Z.); 2 Integral Postgraduate Program in Biotechnology, Faculty of Chemical and Biological Sciences, Autonomous University of Sinaloa, Ciudad Universitaria, Culiacan 80010, Mexico; 3 Faculty of Physical Education and Sports, University of Sinaloa, Culiacan 80013, Mexico; giovanni.ramirez@uas.edu.mx; 4 Department of Engineering, Technological National of Mexico, Technological Institute of the Yaqui Valley, Bacum 85276, Mexico; mgracia.valenzuela@itvy.edu.mx; 5 Postgraduate Program in Health Sciences, Faculty of Biological and Health Sciences, University of Sonora, Hermosillo 83000, Mexico; aldo.arvizu@unison.mx; 6 Clinical and Research Laboratory (LACIUS, U.N.), Department of Chemical, Biological, and Agricultural Sciences (DC-QB), Faculty of Biological and Health Sciences, University of Sonora, Navojoa 85880, Mexico

**Keywords:** food allergy, parent report, anaphylaxis, egg allergy, milk allergy, food allergen, survey

## Abstract

The magnitude and relevance of food allergies in the preschool population remain unknown in most regions of Mexico and Latin America. Thus, our aim was to estimate the parent-reported prevalence of food allergies in a Mexican preschool population and to characterize their clinical diagnosis and presentation. A cross-sectional survey was conducted in Culiacán City. A validated questionnaire was utilized. A total of 810 parents responded to the questionnaire (valid response rate, 40.7%). The estimated prevalence rates (95% CI) were: “physician-diagnosed Food Allergy (FA), ever” 5.30% (3.86–7.08); “immediate-type FA, ever” 2.96% (1.91–4.38); “immediate-type FA, current” 1.60% (0.86–2.73); and food-dependent anaphylaxis 1.11% (0.51–2.01). The main food allergens were milk (0.49%), strawberries (0.37%), egg, and soy (0.25% each). Atopic diseases and a family history of allergies were significantly associated with immediate-type FA. Among “immediate-type FA, current” cases, 76.9% required emergency room visits, but the prescription of epinephrine autoinjectors was reported in one case only. The food reactions occurred at home (92.35%), in a relative’s house (38.5), and at restaurants (23%). Immediate-type FA reactions requiring emergency room visits are not uncommon among the studied population. Actions like proper anaphylaxis management and the prevention of cross-contamination of foods should be encouraged.

## 1. Introduction

IgE-mediated food allergy is an adverse immune reaction triggered by allergenic food proteins [1]. Although non-IgE-mediated allergies are possible, the IgE-mediated one is more relevant due to the quick onset of food allergy symptoms and the possibility of developing life-threatening anaphylaxis [2]. Signs and symptoms of mild to moderate food allergic reactions are more frequently reported than severe ones with anaphylaxis [3]. The prevalence of IgE-mediated food allergies is higher in children than in adults, but the main concern is that the allergic reactions triggered after allergen exposure are far more severe in children aged less than six years than in adults [2,4]. The immune disorder limits the social interaction of the affected individuals, negatively impacting their quality of life and affecting the emotional and economic aspects of caregivers or parents of allergic children [5,6].

Both food allergy prevalence in the child population and the main food triggering it are issues widely addressed in developed countries [7]. Such information has been the basis for proposing and applying public policies aimed at improving the quality of life of allergic individuals [8,9]. Latin America is a developing region that has a population with a huge variety of culinary uses and customs as well as a markedly diverse genetic makeup. These aspects motivate researchers to carry out prevalence studies in specific regions of Latin American countries to characterize the main foods causing allergies in different age groups and to know the magnitude and relevance of the problem. Although there is still limited information [10], most food allergy epidemiological studies carried out in Latin America have been focused on schoolchildren, informing prevalence rates between 1.4 and 5.5% [11,12,13,14,15]. In Mexico, the second-largest Latin American country in terms of population and the third in territorial extension [16], only one food allergy prevalence study has been conducted in preschoolers, which took place in the west of the country [17]. Notably, the populations in the west and the northwest of Mexico are expected to be exposed to a different environment and culinary habits and to carry a distinct genetic makeup [18]. Thus, to expand our knowledge about the prevalence and triggers of food allergies in preschoolers in Latin America, a parent-reported prevalence study was carried out in preschoolers in a city in the northwest of Mexico.

## 2. Materials and Methods

### 2.1. Population Survey

A population-based, cross-sectional survey was conducted among parents of preschoolers (aged 3–6 years old) in Culiacán, Sinaloa, Mexico, from December 2022 to February 2023. Culiacán is an urban settlement and the capital city of the state of Sinaloa in the northwest of Mexico. Different socioeconomic statuses are found among the population of this capital city [19]. The general perception is that families of low to middle or middle to high socioeconomic status are likely to enroll their children in public or private kindergartens, respectively [20]. A simple random sampling was carried out, where each kindergarten was considered a cluster. All parents of the preschoolers were invited to participate in the study. Ten public and seven private kindergartens from Culiacán City were randomly selected from the online database of the National Statistical Directory of Economic Units published by the National System of Statistical and Geographical Information (INEGI) [21] (611122 and 6111221 were the codes to find public and private preschools, respectively). A letter about the study’s methodological characteristics and aim was handed out to the principals of each kindergarten. After obtaining permission, the teachers sent the children’s parents a text message containing the link to the survey. Informed consent was digitally obtained. For this purpose, a summary of the study was provided on the first page of the questionnaire, and only the parents who agreed to participate could answer the survey. Inclusion criteria were as follows: (1) Mexican preschoolers aged three to six years, (2) enrolled in the selected kindergartens, (3) whose parents read and completed the questionnaire by themselves, and (4) provided consent. Questionnaires with incomplete demographic data were excluded.

The sample size was calculated using OpenEpi [22]. A formula for estimating proportions for an infinite population was selected. The following parameters were chosen for sample size estimation: an expected proportion of immediate-type food allergies by parent report of 3.5% [11], 99% confidence, and an absolute error of 2%. A sample size of at least 560 children was considered representative of preschoolers from Culiacán City. The Ethics Review Board of the Faculty of Nutrition and Gastronomy of the Autonomous University of Sinaloa reviewed and approved the study protocol (ethic approval number CE-UACNYG-2014-AGO-001). The ethical guidelines established by the Declaration of Helsinki were taken into account.

### 2.2. Questionnaire

A self-administered questionnaire was adapted and utilized in the present study [11,13]. The questionnaire was digitalized using the SurveyMonkey online platform. The questionnaire collected demographic data about the preschoolers (age, sex, preschool setting) and information about type of delivery, feeding method in the first year, atopic disease history, and family history of atopic conditions.

Parents who reported that their children experienced adverse reactions to food completed an in-depth questionnaire section. This section collected information regarding the specific food(s) triggering the symptoms, the time of the onset of the symptoms after food(s) consumption, symptoms developed, emergency room visits, medical prescriptions to ameliorate the allergic reactions, and recommendations. Additionally, parents gave information about the locations where the allergic reactions occurred, the age of the child when he or she consumed the offending food(s) for the first time, and when the symptoms appeared for the first time. Parents who reported that their child had a medical diagnosis of food allergy were asked about the diagnosis method and the specific involvement in the health area of the professional who made the diagnosis.

### 2.3. Classification of Individuals

The individuals were classified as follows [11,12,15]: (1) Parent-reported physician-diagnosed food allergy (PR-PD FA), preschoolers whose parents reported medical diagnosis of food allergy; (2) “Immediate-type FA, ever”, preschoolers whose parents reported recurrent and convincing symptoms of immediate-type food allergy; (3) “Immediate-type FA, current”, preschoolers that met criteria for “immediate-type FA, ever”, but whose parents reported that, currently, their children are unable to eat the suspect food without triggering any reaction; (4) “Food-dependent anaphylaxis”, according to [15].

Convincing symptoms of an immediate-type food allergy were considered when urticaria, angioedema, trouble breathing, wheezing, throat tightness, vomiting, or diarrhea were reported to occur within the next 2 h after the offending food was ingested.

### 2.4. Statistical Analysis

Categorical and continuous variables were described as total numbers and percentages. A two-tailed Fisher’s exact test was used to compare proportions between groups and determine associations. The associations are presented as odds ratios (OR) with 95% confidence intervals (CI) calculated in a basic 2 × 2 table. The statistical software GraphPad Prism Version 9.3 (GraphPad Software, San Diego, CA, USA) was used for statistical analyses. The OpenEpi software version 3.03a (Atlanta, GA, USA) was used to estimate the prevalence rates (reported per 100 inhabitants, 95% IC).

## 3. Results

### 3.1. Participants and Demographic Characteristics

A total of 1990 parents was invited to participate in the survey. Among these, 855 accepted to participate in the study (response rate, 44.96%). Forty-five questionnaires were excluded due to incomplete demographic data. Thus, 810 questionnaires were considered for prevalence rates estimations (valid response rate, 40.7%). Among participants, 54.2% (n = 439) were male, and 77.53% (n = 628) of the children were enrolled in public kindergartens (Table 1). A total of 329 (40.61%) preschoolers had a diagnosis of allergy disease, and 141 (17.4%) had more than one allergic condition. Allergic rhinitis (17.65%) and atopic dermatitis (14.32%) were the most reported atopic diseases (Table 1).

### 3.2. Prevalence Rates Estimations

Table 2 shows the prevalence rate estimations. A total of 60 parents reported that their children experienced adverse reactions after consuming specific foods. However, only 24 reported a history of convincing symptoms of an immediate-type FA reaction (immediate-type FA, ever: 2.96%, 95% CI, 1.91–4.38). Of these cases, 11 preschoolers had outgrown their food allergy, as they are currently able to consume the suspected food without triggering symptoms. Therefore, the prevalence of immediate-type FA current was 1.60% (n = 13) (95% CI, 0.86–2.73). Among these allergic children, nine developed allergic manifestations compatible with an anaphylactic reaction (Food-induced anaphylaxis: 1.11%, 95% CI, 0.51–2.01). No statistical differences were observed by sex or type of kindergarten (*p* > 0.05, Supplementary Material, Tables S1 and S2).

### 3.3. Food Allergens and Clinical Characteristics of FA Reactions

**Figure 1:**
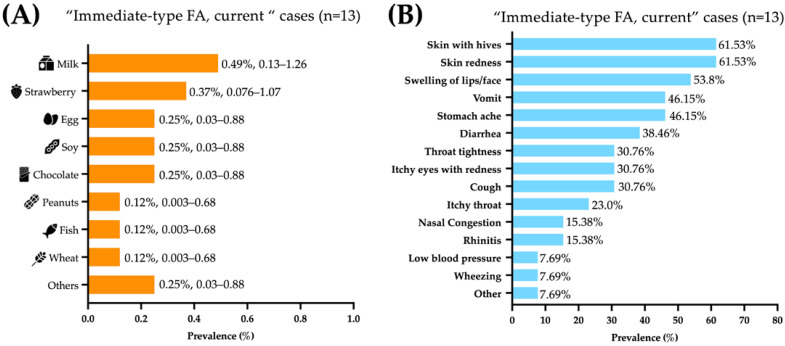
Foods and symptoms associated with “immediate-type food FA, current”. (**A**) Food allergy prevalence by specific food, presented as percentage and 95% confidence intervals. (**B**) frequency of specific symptoms in Mexican preschoolers with “immediate-type FA, current” (n = 13).

The foods triggering food allergic reactions were (prevalence, 95% CI): milk (0.49%, 0.134–1.259), strawberries (0.37%, 0.076–1.078), and egg, soy, and chocolate (0.25%, 0.0303–0.888 each) (Figure 1A). The immediate-type food allergic reactions mainly affected the skin (61.5%), gastrointestinal (61.5%), and respiratory tracts (46.25%). Most symptoms reported were skin with hives (61.53%), skin redness (61.53%), swelling of lips/face (53.84%), vomit (46.15%), abdominal pain (46.15%), and diarrhea (38.46%) (Figure 1B).

Among the “immediate-type FA, current” cases, 76.9% (10 out of 13) received medical attention in the emergency room for symptoms triggered after food consumption. Antihistamines (55.5%) were the main treatment used for food allergic reactions. Surprisingly, only one parent reported that their child was prescribed an epinephrine auto-injector. Regarding places where the food reactions took place, 92.3% of immediate-type FA cases occurred at home, 38.5% in a relative´s house, and 23% in a restaurant. Interestingly, in 90% of the immediate-type FA cases the consumption of the offending food occurred for the very first time after one year of age.

### 3.4. Risk Factors Associated with Immediate-Type FA Cases

The presence of other atopic diseases such as chronic urticaria (OR 12.65, 95% IC 5.262–30.24), animal allergies (OR 9.107, 95% IC 3.538–23.29), allergic rhinitis (OR 7.774, 95% IC 3.532–17.02), insect sting allergy (OR 6.778, 95% IC 2.875–16.05), atopic dermatitis (OR 6.558, 95% IC 2.796–15.29), allergic conjunctivitis (OR 5.180, 95% IC 2.016–14.51), and drug allergy (OR 3.073, 95% IC 1.21–8.219) was significantly associated with immediate-type FA cases (n = 24) (percentages and *p* values are shown in Supplementary Material Table S3). No significant associations were found between the type of delivery or the type of feeding method and the development of immediate-type FA (percentages and *p* values are shown in Supplementary Material, Tables S4 and S5).

A family history of allergic diseases was correlated with the development of immediate-type FA. A history of allergies in the father, including animal allergies (OR 12.66, 95% IC 4.660–37.89), atopic dermatitis (OR 6.057, 95% IC 2.332–15.78), allergic rhinitis (OR 5.32, 95% IC 2.335–12.01), allergic conjunctivitis (OR 5.204, 95% IC 1.53–18.22), and insect sting allergy (OR 5.04, 95% IC 1.95–13.21) was significantly associated with immediate-type FA (percentages and *p* values are shown in Supplementary Material Table S6).

Similarly, a maternal history of insect sting allergy (OR 11.58, 95% IC 4.181–29.95), food allergy (OR 6.624, 95% IC 2.561–16.34), allergic conjunctivitis (OR 6.35, 95% IC 2.196–18.83), atopic dermatitis (OR 5.47, 95% IC 2.104–13.29), allergic rhinitis (OR 5.14, 95% IC 2.339–11.66), animal allergy (OR 4.61, 95% IC 1.807–11.72), and drug allergy (OR 4.07, 95% IC 1.771–9.353) was also significantly associated with immediate-type FA cases (percentages and *p* values are shown in Supplementary Material Table S7). On the other hand, having a sibling with a history of animal allergy (OR 5.72, 95% IC 1.95–17.93), atopic dermatitis (OR 4.39, 95% IC 1.67–12.03), and allergic rhinitis (OR 3.25, 95% IC 1.228–8.259) was significantly associated with immediate-type FA cases (percentages and *p* values are shown in Supplementary Material Table S8).

### 3.5. Characteristics of Physician Diagnosis of FA Cases

**Figure 2:**
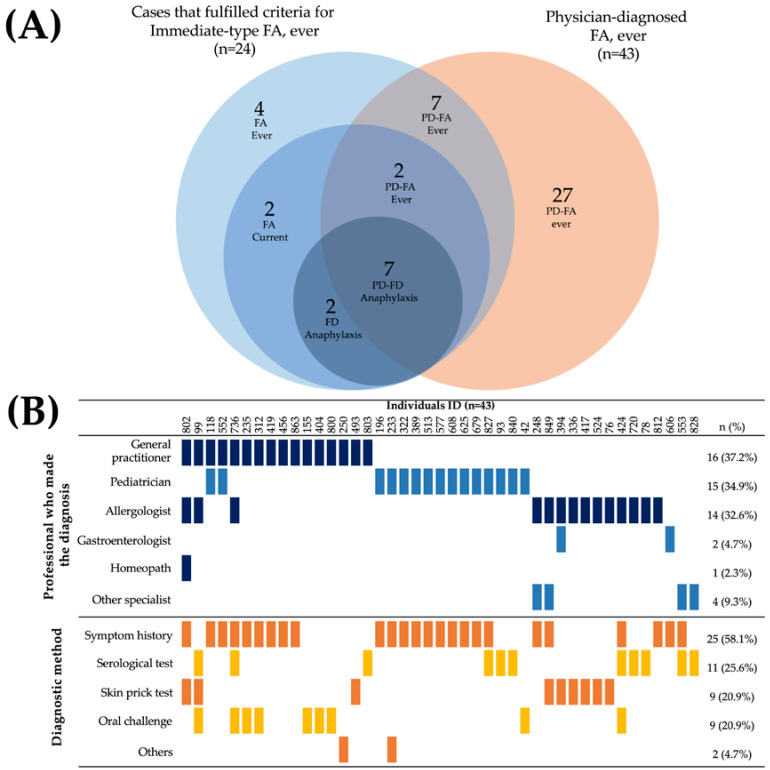
Characteristics of parent-reported physician-diagnosed food allergy cases. (**A**) Overlap of physician-diagnosed food allergy cases and individuals that fulfilled criteria for “Immediate-type FA, ever”. (**B**) Health professionals who made the diagnosis of food allergy and diagnostic method.

A total of 43 parents reported that their children had a medically diagnosed food allergy (physician-diagnosed FA 5.30%, CI 95%: 3.86–7.08). However, only 16 of these preschoolers had a history of symptoms characteristic of an immediate-type food allergy reaction, of whom 9 had not yet outgrown their food allergy (Figure 2A). The majority of cases (7 out of 9) that met the criteria for “food-dependent anaphylaxis” had a physician diagnosis of FA. The food allergy diagnoses were mainly made by general physicians (37.2%), pediatricians (34.9%), and allergologists (32.6%). Diagnosis was primarily based on the clinical history focused on the symptoms (58.1%), either as the unique diagnostic method (37.2%, n = 16) or in combination with an objective test (20.93%, n = 9) (Figure 2B). Surprisingly, only 20.9% of children with a medical diagnosis of food allergy underwent an oral challenge to confirm the diagnosis (9 out of 43). Thirteen out of fourteen allergologists involved in the diagnosis of food allergies carried out objective tests to make the diagnosis.

## 4. Discussion

The estimated parent-reported prevalence of “immediate-type FA, current” in Mexican schoolchildren from Culiacán, a city in the northwest of Mexico, was 1.6%. This estimation falls within the range of most survey-based prevalence rates reported in Asia, Europe, North America, and Latin America (from 0.4% to 17.6%) [17,23,24,25,26,27,28,29,30,31,32,33,34]. Notably, the prevalence rate estimated in the present study is lower than the one reported for preschoolers from Guadalajara (4.1%), a city in the west of Mexico. Beyond food allergy definitions, these different prevalence rate estimations could be explained by the fact that Mexico, with a territorial area of 1,960,189 km^2^, features a wide variety of climates (warm, dry, and temperate) and diverse ecosystems (deserts, forests, mountains, and reefs), which can determine the availability and accessibility of different types of food, influencing dietary habits [16,35]. Furthermore, the distinct genetic makeup inherent to the Mexican population [18], socioeconomic aspects, and feeding patterns, especially in the first year of life, may also contribute to such a difference. Therefore, estimations of food allergy prevalence rates, identification of the primary triggers, and characterization of the clinical manifestations across various geographical regions are desirable to establish the magnitude and relevance of the problem.

The most frequently reported allergenic foods were milk (0.49%), strawberries (0.37%), and eggs (0.25%). This is in line with the most frequently reported allergenic foods in preschoolers in European, Asian, and Latin American countries [25,33,36,37,38,39]. In fact, the only study carried out in a Mexican preschool population reported that milk (1.0%) and fruits (1.0%) were the most common triggers of allergic reactions among potential food allergic children from a city (Guadalajara) in the west of Mexico [17]. Our research group reported similar results in a survey carried out among schoolchildren aged 5–13, in the same geographical region and using the same questionnaire (egg = 0.40%; milk = 0.29%) [11]. It should be highlighted that although egg and milk allergies are among the most frequently reported food allergies in preschoolers, they are usually outgrown in childhood [40]. In this regard, IgE sensitization to eggs and milk is more frequent in children younger than five years than in older ones and adults [41]. The molecular mechanisms involved in transitory and persistent food allergies are not well understood. Certainly, the production of highly specific IgE against egg allergens and differences in the microbiome are some features associated with persistent food allergies [42]. It is important to recognize that strawberries have a histamine content that could induce symptoms similar to allergies in individuals who are not sensitized [43,44]. However, since IgE sensitization to strawberries has been reported [41], carrying out diagnostic assessments in individuals suspected of a strawberry allergy should be encouraged.

Cesarean delivery [45], the type of lactation during the first months of life [46], a diagnosis of other atopic diseases [47], and a family history of allergic diseases [48] are risk factors associated with the development of food allergies. Consistent with a recent meta-analysis [49], the present study found significant associations between food allergy development and chronic urticaria, allergic rhinitis, atopic dermatitis, and allergic conjunctivitis. The “atopic march” theory has been proposed to describe the progression and interrelationships of these atopic diseases [50]. This concept postulates a typical progression of allergic diseases that often have their onset early in life with atopic dermatitis, followed in some cases by food allergies, allergic rhinitis, and asthma. Moreover, our study found that a parental history of allergic disease, either in the mother or father, is significantly associated with immediate-type food allergy development, emphasizing the role of the genetic makeup in the development of food allergy [51]. This last point highlights the need to promote early intervention strategies for preventing and managing food allergies, especially in families with a history of allergic diseases.

Anaphylaxis is a systemic allergic reaction characterized by a rapid onset of signs and symptoms that may potentially cause death. There has been an increase of 377% (from 2007 to 2016) in clinical procedures involved in the management or diagnosis of food-dependent anaphylaxis in the United States of America [52], which highlights that the condition could be growing in prevalence. In the present study, more than half of “immediate-type FA, current” cases (76.9%) received medical attention because of the allergic symptoms they developed. Although the first-line treatment of life-threatening anaphylaxis is the early administration of intramuscular epinephrine/adrenalin [53], an epinephrine auto-injector was prescribed in only one out of nine cases of anaphylaxis in the present study. The scarce prescription of epinephrine auto-injectors has been reported in other Latin American countries [11,12,15]. The recent publication of guidelines for the management of anaphylaxis [54] and the low availability of auto-injectors in mainstream drugstores in Latin America could influence the very low prescription of epinephrine devices. In this context, a study carried out in Mexico shows that only 49.5% of physicians recommend the use of intramuscular epinephrine as a first-line treatment during an anaphylactic reaction, highlighting a possible underuse of epinephrine [55].

Food-dependent anaphylactic episodes commonly occur at home or in restaurants [56]. In this context, most immediate-type allergic reactions reported in the present study were triggered at home, in a relative´s house, or in restaurants. Therefore, based on the present data and available literature, the implementation of programs for disseminating clinical guidelines for the proper management of anaphylaxis and for preventing cross- contamination of foods in restaurants, as well as encouraging adherence to regulations that facilitate alerting consumers about allergen content in food products, are actions of relevance not only in the northwest of Mexico but also in other Latin American regions.

The gold standard for food allergy diagnosis is a single- or double-blind placebo-controlled food challenge [57]. However, food challenges are stressful for patients, and special clinical settings and trained personnel are required to perform them. Alternatively, the combination of a convincing clinical history and positive diagnostic tests (e.g., specific IgE serum levels and skin prick test) can be employed to establish a diagnosis of food allergy [58]. In the present study, some physicians (37.2%) made the diagnosis of food allergy based only on the history of the symptoms triggered after food consumption. The clinical manifestations of food allergies are highly diverse, and performing objective diagnostic tests is highly desirable to establish a food allergy diagnosis [59].

The main strengths of our study are its population-based design, which includes children with different socioeconomic status and from different geographical areas of Culiacán City. Private and public schools commonly enroll students from low- to middle- or middle- to high-socioeconomic status families in Latin America, respectively [20]. An additional strength is the collection of information about the diagnostic approach and the health professional who made the diagnosis of food allergy. We should acknowledge that our study is not devoid of limitations. Firstly, incorporating data from other geographical regions of Mexico would allow us to establish the different profiles of foods that trigger allergic reactions in Mexican preschoolers. Secondly, the relatively low response rate (44.96%) could influence the prevalence rate estimations. And thirdly, the food allergy cases identified for prevalence rate estimations were based on parental reports only and were not confirmed with objective diagnostic tests such as skin prick tests, specific IgE serological tests, or food challenges. Certainly, the parents were aware that the study was focused on evaluating the prevalence of food allergies. This fact could be a potential source of bias because families with a food-allergic child may have been more prompted to complete the survey than families without a food-allergic child, which could lead to an overestimation of the prevalence rates. These potential biases should be considered when interpreting the prevalence rate estimations informed by the present study. Despite limitations, our study generated relevant data about the prevalence and management of food allergies among preschoolers, a population lacking epidemiologic food allergy data in the northwest of Mexico. Finally, the present study serves as the groundwork for further research based on objective diagnostic criteria.

## 5. Conclusions

Immediate-type FA reactions requiring emergency room visits are not uncommon among preschoolers from the northwest of Mexico. The prevalence rate of food allergies is 1.6%, with milk, strawberries, and eggs as the main triggers of allergic reactions. Notably, most allergic cases met the characteristics of anaphylaxis, occurring at home or in restaurants. These findings highlight that actions for the proper management of severe allergic reactions and avoiding cross-contamination of foods are desirable. Finally, the association between a parental history of allergic disease and the development of immediate-type food allergies is a topic that deserves further in-depth studies to establish the need for programs for preventing food allergies.

## Figures and Tables

**Table 1:** Demographic characteristics of the study population.

Mean age in years (range)		4.51 (3–6)
**Variable**		**n (%)**
Sex	Female	371 (45.8)
	Male	439 (54.2)
Preschooler	Public	628 (77.53)
	Private	182 (22.46)
**Known allergic disease**		**n (%)**
Allergic rhinitis		143 (17.65)
Atopic dermatitis		116 (14.32)
Drug allergy		67 (8.27)
Insect sting allergy		62 (7.65)
Asthma		61 (7.53)
Chronic urticaria		52 (6.42)
Conjunctivitis		43 (5.31)
Animals’ allergy		41 (5.06)
Anaphylaxis		2 (0.25)

**Table 2:** Prevalence estimations.

Assessment	Number of Cases	Prevalence % (95% CI) Total, N = 810
Adverse food reactions	60	7.40 (5.7–9.43)
Perceived FA, ever	93	11.48 (9.36–13.88)
Physician-diagnosed FA, ever	43	5.30 (3.86–7.08)
Immediate-type FA, ever	24	2.96 (1.91–4.38)
Immediate-type FA, current	13	1.60 (0.86–2.73)
Food-induced anaphylaxis	9	1.11 (0.51–2.01)

## Data Availability

The data presented in this study are available on request from the corresponding author.

## Supplementary Materials

The following supporting information can be downloaded at: https://www.mdpi.com/article/10.3390/jcm12155095/s1, Table S1: Prevalence estimations by sex; Table S2: Prevalence estimations by type of preschool; Table S3: Associations between other allergic diseases diagnosis and immediate-type food allergy development; Table S4: Type of delivery and its association with the risk of immediate-type food allergy development; Table S5: Feeding method during the first year and association with the risk of immediate-type food allergy development; Table S6: Association between father’s history of allergic diseases and immediate-type food allergy development; Table S7: Association between mother´s history of allergic diseases and immediate-type food allergy development; Table S8: Association between sibling’s history of allergic diseases and immediate-type food allergy development.
