# Supplementary material for: Prevalence of Parent-Reported Food Allergy in a Mexican Pre-School Population
Source: J Clin Med. 2023 Aug 3;12(15):5095. doi: 10.3390/jcm12155095 (PMC10420166; doi:10.3390/jcm12155095)
Supplement: Supplementary file 1 [file jcm-12-05095-s001.zip › jcm-2522941-supplementary.pdf]

Food Allergy Prevalence in Mexican Preschoolers Estimated by Parent-Report

Supplementary Materials

Table S1. Prevalence estimations by type of sex

Assessment	Number of reported cases	Prevalence % (95% CI)		p
		Male=422	Female=547	
Adverse food reactions	60	7.97 (5.61–10.91)	6.73 (4.40–9.78)	0.5905
Perceived FA, ever	93	12.52 (9.57–15.99)	10.24 (7.35–13.79)	0.3217
Physician-diagnosed FA, ever	43	5.92 (3.90–8.55)	4.58 (2.69–7.23)	0.4345
Immediate-type FA, ever	24	2.73 (1.42–4.72)	3.23 (1.68–5.58)	0.6836
Immediate-type FA, current	13	1.59 (0.64–3.25)	1.61 (0.59–3.48)	>0.9999
Food-induced anaphylaxis	9	1.13 (0.37–2.63)	1.07 (0.29–2.73)	>0.9999

Table S2. Prevalence estimations by type of preschool

Assessment	Number of reported cases	Prevalence % (95% CI)		*p*
		Public=628	Private=182	
Adverse food reactions	60	7.32 (5.41–9.65)	7.69 (4.26–12.57)	0.8726
Perceived FA, ever	93	10.35 (8.08–13.00)	15.38 (10.47–21.46)	0.0652
Physician-diagnosed FA, ever	43	4.93 (3.37–6.93)	6.59 (3.45–11.23)	0.3544
Immediate-type FA, ever	24	3.18 (1.95–4.87)	2.19 (0.60–5.53)	0.6239
Immediate-type FA, current	13	1.75 (0.87–3.11)	1.09 (1.13–3.91)	0.7433
Food-induced anaphylaxis	9	1.27 (0.55–2.49)	0.54 (0.01–3.02)	0.6922

Table S3. Association between other allergic diseases diagnosis and immediate-type food allergy development.

Allergic disease	Immediate-type FA, ever (n=24) n (%)	Non-FA (n=786) n (%)	p	*Odds Ratio (95% CI)*
Asthma	4 (16.66)	57 (7.25)	0.0991	2.558 (0.9166–7.560)
Chronic urticaria	10 (41.66)	42 (5.34)	**<0.0001**	12.65 (5.262–30.24)
Allergic rhinitis	15 (62.5)	128 (16.28)	**<0.0001**	7.774 (3.532–17.02)
Anaphylaxis	–	2 (0.25)	>0.9999	–
Atopic dermatitis	12 (50.00)	104 (13.23)	**<0.0001**	6.558 (2.796–15.29)
Insect sting allergy	8 (33.33)	54 (6.87)	**0.0002**	6.778 (2.875–16.05)
Animals allergy	7 (29.16)	34 (4.32)	**<0.0001**	9.107 (3.538–23.29)
Allergic conjunctivitis	5 (20.83)	38 (4.83)	**0.0067**	5.180 (2.016–14.51)
Drug allergy	5 (20.83)	62 (7.88)	**0.0411**	3.073 (1.216–8.219)

Table S4. Type of delivery and its association with the risk of immediate-type food allergy development.

Allergic disease	Caesarean section (n=511) n (%)	Vaginal birth (n=299) n (%)	p	*Odds Ratio (95% CI)*
Immediate-type FA, ever	15 (2.93)	9 (3.01)	>0.9999	0.9745 (0.4434–2.337)

Table S5. Feeding method during the first year of life and its association with the risk of immediate-type food allergy development.

Allergic disease	Non-exclusive breastfeeding (n=609) n (%)	Exclusive breastfeeding (n=201) n (%)	p	*Odds Ratio (95% CI)*
Immediate-type FA, ever	16 (2.62)	8 (3.98)	>0.3396	0.6509 (0.2906–1-461)

Table S6. Association between Father's History of Allergic Diseases and Immediate-Type Food Allergy Development.

**Allergic disease**	**Immediate-type FA, ever (n=24)** **n (%)**	**Non-FA (n=786)** **n (%)**	**p**	***Odds Ratio (95% CI)***
Asthma	3 (12.5)	27 (3.43)	0.0549	4.016 (1.199–13.41)
Food allergy	3 (12.5)	31 (3.94)	0.0748	3.479 (1.046–11.38)
Allergic rhinitis	10 (41.66)	93 (11.83)	0.0003	5.323 (2.335–12.01)
Anaphylaxis	–	2 (0.25)	–	–
Atopic dermatitis	6 (25.00)	41 (5.21)	0.0017	6.057 (2.332–15.78)
Insect sting allergy	5 (20.83)	16 (2.03)	0.0077	5.04 (1.95 – 13.21)
Animals allergy	6 (11.76)	17 (2.23)	0.0002	12.66 (4.660–37.89)
Allergic conjunctivitis	3 (12.5)	21 (2.67)	0.0307	5.204 (1.533–18.22)
Drug allergy	3 (4.16)	45 (5.72)	>0.9999	0.7159 (0.06764–4.060)

Table S7. Association between Mother´s History of Allergic Diseases and Immediate-Type Food Allergy Development.

**Allergic disease**	**Immediate-type FA, ever (n=24)** **n (%)**	**Non-FA (n=786)** **n (%)**	**p**	***Odds Ratio (95% CI)***
Asthma	2 (8.33)	30 (3.81)	0.2442	2.291 (0.5110–9.507)
Food allergy	7 (29.16)	46 (5.85)	**0.0005**	6.624 (2.561–16.34)
Allergic rhinitis	11 (45.83)	111 (14.12)	**0.0003**	5.146 (2.339–11.66)
Anaphylaxis	–	1 (0.12)	–	–
Atopic dermatitis	7 (29.16)	55 (6.99)	**0.0013**	5.473 (2.104–13.29)
Insect sting allergy	6 (25.00)	22 (2.79)	**<0.0001**	11.58 (4.181–29.95)
Animals allergy	6 (25.00)	53 (6.74)	**0.0055**	4.610 (1.807–11.72)
Allergic conjunctivitis	4 (16.6)	24 (3.05)	**0.0075**	6.350 (2.196–18.83)
Drug allergy	8 (33.33)	86 (10.94)	**0.0037**	4.070 (1.771–9.353)

Table S8. Association between Sibling's History of Allergic Diseases and Immediate-Type Food Allergy Development.

**Allergic disease**	**Immediate-type FA, ever (n=19)**<br>**n (%)**	**Non-FA (n=652)**<br>**n (%)**	**p**	***Odds Ratio (95% CI)***
Asthma	3 (15.78)	31 (4.75)	0.066	3.756 (1.108–13.24)
Food allergy	3 (15.78)	35 (5.36)	0.0866	3.305 (0.9796–11.48)
Allergic rhinitis	6 (31.57)	81 (12.42)	**0.0267**	3.254 (1.228–8.259)
Anaphylaxis	–	1 (0.15)	–	–
Atopic dermatitis	5 (26.31)	49 (7.51)	**0.0136**	4.395 (1.679–12.03)
Insect sting allergy	1 (5.26)	20 (3.06)	0.458	1.756 (0.1602–11.53)
Animals allergy	4 (21.05)	29 (4.44)	**0.0112**	5.729 (1.956–17.93)
Allergic conjunctivitis	–	14 (2.14)	–	–
Drug allergy	3 (15.78)	49 (7.51)	0.1765	2.307 (0.6913–7.755)
